# Incomplete homogenization of 18 S ribosomal DNA coding regions in *Arabidopsis thaliana*

**DOI:** 10.1186/1756-0500-4-93

**Published:** 2011-03-31

**Authors:** Ayalew B Mentewab, Megan J Jacobsen, Rebekah A Flowers

**Affiliations:** 1 Biology Department, Spelman College, 350 Spelman Lane, Atlanta, GA 30314, USA; 2 Biology Department, Emory University, 1510 Clifton Road, Atlanta, Georgia 30322, USA; 3 ChemRisk, LLC, 101 2nd Street, suite 700, San Francisco, CA 94105, USA

## Abstract

**Background:**

As a result of concerted evolution, coding regions of ribosomal DNA sequences are highly conserved within species and variation is generally thought to be limited to a few nucleotides. However, rDNA sequence variation has not been systematically examined in plant genomes, including that of the model plant *Arabidopsis thaliana *whose genome was the first to be sequenced.

**Findings:**

Both genomic and transcribed 18 S sequences were sampled and revealed that most deviation from the consensus sequence was limited to single nucleotide substitutions except for a variant with a 270 bp deletion from position 456 to 725 in *Arabidopsis *numbering. The deletion maps to the functionally important and highly conserved 530 loop or helix18 in the structure of *E. coli *16 S. The expression of the deletion variant is tightly controlled during developmental growth stages. Transcripts were not detectable in young seedlings but could be amplified from RNA extracts of mature leaves, stems, flowers and roots of *Arabidopsis thaliana *ecotype Columbia. We also show polymorphism for the deletion variant among four *Arabidopsis *ecotypes examined.

**Conclusion:**

Despite a strong purifying selection that might be expected against functionally impaired rDNAs, the newly identified variant is maintained in the *Arabidopsis *genome. The expression of the variant and the polymorphism displayed by *Arabidopsis *ecotypes suggest a transition state in concerted evolution.

## Background

In eukaryotes, the 18 S, 5.8 S, and 25 S rRNAs are encoded as a single transcript from rDNA repeats, arranged in head to tail arrays and separated by spacer regions. Within a given species, rDNA repeats are often identical, which has lead to the proposal that rDNA loci undergo a concerted evolution [1,2]. Concerted evolution results in rapid horizontal homogenization of a select variant through a number of molecular processes such as unequal crossing over and gene conversion. Additionally, functionally constrained regions such as those encoding the 18 S and 25 S genes are subject to a strong purifying selection resulting in a high degree of conservation across species [3].

Nevertheless, an increasing number of studies motivated by phylogenetic analysis have uncovered the presence of divergent rDNA paralogs and pseudogenes in a number of taxa [4-7]. Systematic studies on the extent and nature of sequence variation have been limited. Indeed, in fully sequenced genomes, regions of rDNA repeats cannot be tiled into contigs and thus megabase size gaps remain unassembled. In *Drosophila *and several fungal genomes rDNA variability was examined using traces from whole-genome shotgun sequencing projects [8-11]. All four studies point to the existence of a small degree of polymorphism among rDNA repeats, with the vast majority of polymorphic sites located in the spacer regions. For example, out of 227 polymorphic sites detected in several yeast strains, only 44 sites mapped to the rRNA-encoding genes [11]. Within the coding regions, polymorphisms were three to eight times more frequent in the expansion segments compared with the conserved core regions that are functionally constrained.

To date, sequence level variation in *Arabidopsis *rDNA coding regions remains unexplored. The BAC-end sequencing approach taken for the *Arabidopsis thaliana *genome precludes the use of trace sequences to examine sequence variation [12]. There are approximately 1200-1500 repeats per diploid genome, at the tips of chromosomes 2 and 4, with two rDNA arrays that could be distinguished by RFLP analysis [12,13]. A single complete rDNA unit is also found in the centromeric region of chromosome 3. Here we report on the presence of a 270 bp deletion variant of 18 S rDNA that suggests *Arabidopsis *rDNA arrays are in transition stages of concerted evolution.

## Results

**Figure 1:**
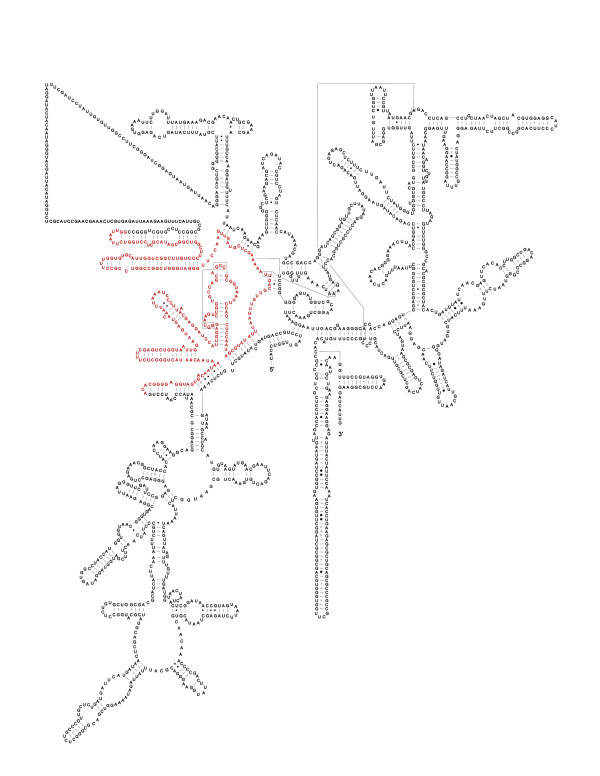
**Location of the 270 bp deletion on the secondary structure of *Arabidopsis *18 S RNA**. The missing fragment in the newly identified variant is highlighted in red on the secondary structure of *Arabidopsis *18 S RNA

BAC-end sequencing of the *Arabidopsis *genome precludes direct analysis from trace sequences, thus to sample sequence variation of 18 S rDNA, genomic DNA was amplified. A total of 47 individual clones were generated. After sequence assembly with CAP3, two contigs were identified with 44 clones in the first contig and 3 clones in the second contig. The first contig was identical to the 18 S sequence from At3g41768 (GenBank ID: 186510611) and the vast majority of the clones fell in that contig group. Among those 44 clones, sequence variation was very limited, with only a total of 6 clones showing single nucleotide polymorphisms (Table 1). The level of polymorphism captured is 0.3% per fragment length and 13.6% per sequenced clones. Thus sequence analysis limited to the first contig supports the view that rDNA sequences are overall highly homogenous. However, the second contig identified, drastically deviated from the canonical sequence. It lacked a 270 bp fragment corresponding to positions 456-725 in the canonical 18 S sequence (Figure 1). The deletion encompasses the functionally important helix18 critical for ribosome function. In addition, a A151C mutation was also found to be characteristic of the second contig. BLAST searches were carried out to identify similar variants in GenBank but none were detected. Thus the 270 bp deletion variant was new and unusual. A total of 3 clones, obtained from 2 independent samples fell in the second contig group. The deleted variant was thus detected in this experiment at a frequency of 6.4%.

**Table 1:** Positions and bases found to deviate from the consensus sequence (At3g41768) among clones obtained from the amplification of *Arabidopsis *genomic DNA falling within contigs 1 and 2.

Nucleotide positions	consensus	Contig1 clones	Contig2 clones
87	A	C	

151	A		C (3)

347	G	A	

388	G	T	

456-725			- (3)

642	G	A	

1253	A	G	

1315	C	T	

# clones deviating from consensus		6	3

Total clones		44	3

**Figure 2:**
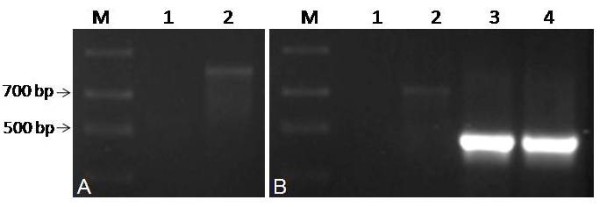
**PCR detection of 18 S variant with 270 bp deletion**. *Arabidopsis *genomic DNA was PCR amplified with primer pairs for the detection of (A) the internal control eIF4A (lanes and templates: M: Marker, 1: water, 2: *Arabidopsis *genomic DNA) and (B) the deleted 18 S (lanes and templates: M: ladder, 1: water, 2: full length 18 S plasmid (negative control), 3: deleted 18 S plasmid (positive control), 4: *Arabidopsis *genomic DNA. Arrows indicate 500 and 700 bp bands from ladder.

To confirm that the observed deletion is not a PCR or cloning artifact, we directly amplified the deleted variant from genomic DNA using a new reverse primer. The new reverse primer was designed such that the primer overlapped the deleted region and would only anneal to the new variant. PCR was carried out with a polymerase lacking proofreading activity. Plasmids with the full length and deleted 18 S variants served as negative and positive controls. A bright band at 465 bp was consistently obtained from the deleted variant while no visible band or a faint band of 730 bp was amplified from the full length 18 S plasmid. As shown in Figure 2, the expected 465 bp fragment was generated from *Arabidopsis *genomic DNA. The PCR product was further sequenced to ensure an 18 S fragment was amplified. These results confirm the presence of a new rDNA variant with 270 bp deletion in the *Arabidopsis *genome.

To examine whether particular sequences were enriched in the transcribed pool of 18 S genes, total RNA was extracted, DNAseI treated and reverse transcribed. PCR was performed on cDNA, 18 S fragments cloned, and a total of 34 clones were sequenced. All sequences aligned with the consensus, however a high number of polymorphism was observed (Table 2). Indeed deviation from the consensus sequence was observed at 30 different positions, with most clones having single nucleotide polymorphisms. The level of polymorphism is 1.7% per fragment length, and 88.2% per sequenced clones, thus about 6 fold higher than that observed for rDNA. As shown in Table 3, the majority of polymorphisms correspond to transitions (76.7%) with T/C transitions being the most frequent (43.3%).

**Table 2:** Positions and bases found to deviate from the consensus sequence (At3g41768) are shown for clones obtained by amplification of *Arabidopsis *cDNA.

Position	Consensus	rRNA
75	T	C

109	A	G

119	T	C

213	T	C

245	T	G

267	G	T

303	A	G

489	C	T

582	T	A

627	A	-

676	C	T

710	G	A

714	T	C (2)

715	T	C

771	C	T

870	G	T

904	A	T

1015	C	A

1098	A	G

1111	G	C

1125	C	T

1133	T	C

1161	C	T

1195	T	C

1198	A	-

1277	G	A

1303	G	A

1390	A	T

1459	T	C

1702	G	C

# clones deviating from consensus		30

total # clones		34

**Table 3:** Frequency of transitions, transversions and indels observed in 18 S rDNA and transcribed rRNA sequences.

Polymorphisms	Transitions								Trans- versions	%	Indels	%	n
	T/C	%	A/G	%	T/G	%	A/C	%					
rDNA	1	12.5	3	37.5	1	12.5	2	25.0	0	0.0	1	12.5	47
rRNA	13	43.3	6	20.0	3	10.0	1	3.3	5	16.7	2	6.7	34

n indicates total number of clones.

**Figure 3:**
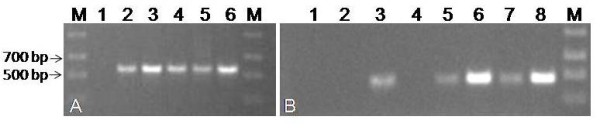
**Expression of 18 S variant with 270 bp deletion**. cDNA from *Arabidopsis *was PCR amplified with primer pairs for the detection of (A) the internal control eIF4A (lanes and cDNA templates: M: Marker, 1: water, 2: young seedlings, 3: flowers, 4: leaves, 5: stems, 6: roots) and (B) the deleted 18 S (lanes and cDNA templates: M: ladder, 1: water, 2: full length 18 S plasmid (negative control), 3: deleted 18 S plasmid, 4: young seedlings, 5: flowers, 6: leaves, 7: stems, 8: roots). Arrows indicate 500 and 700 bp bands from ladder.

The deleted variant was not found among the 34 rRNA clones, raising the question of its expression. We sought to characterize its expression in roots, leaves, stems and flowers of WT *Arabidopsis*. RT-PCR was performed after treatment of the RNA extracts with DNAseI to prevent any amplification from genomic DNA. Lack of contamination with genomic DNA was verified by the sole amplification of a 560 bp fragment from the internal control eIF4A. The deleted variant was detected in mature roots, leaves, stems and flowers but not in young seedlings (Figure 3). Thus the deleted variant is transcribed throughout the plant, except in young seedlings suggesting developmental control of expression.

**Figure 4:**
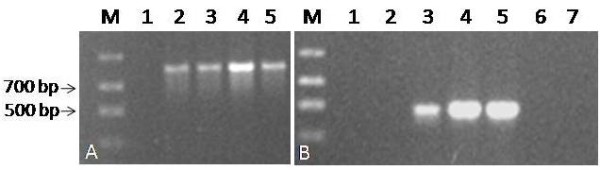
**Polymorphism of *Arabidopsis *accessions for the presence of the deletion variant of 18S**. Genomic DNA from four ecotypes was PCR amplified with primer pairs for the detection of (A) the internal control eIF4A (lanes and templates: M: Marker, 1: water, 2: Columbia, 3: Landsberg erecta, 4: Bay, 5: Shahdara) and (B) the deleted 18 S (lanes and templates: M: ladder, 1: water, 2: full length 18 S plasmid (negative control), 3: deleted 18 S plasmid (positive control) 4: Columbia, 5: Landsberg erecta, 6: Bay, 7: Shahdara). Arrows indicate 500 and 700 bp bands from ladder.

To examine whether the 270 bp deletion is unique to *A. thaliana *ecotype Columbia, we tested for its presence in 3 other ecotypes: Landsberg erecta, Bay and Shahdara. The deletion variant was found in Landsberg erecta, but not Bay and Shahdara indicating polymorphism in *Arabidopsis *populations (Figure 4).

## Discussion and Conclusion

Systematic analysis of sequence variation in 18 S rDNA of *Arabidopsis thaliana *revealed that overall sequences were highly homogenous except for the 270 bp deletion variant. More single nucleotide variants were observed in rRNA sequences, most likely due to the fidelity of reverse transcriptase. The discovery of a 270 bp deletion variant highlights the poor characterization of rDNA variants in sequenced genomes. This variant was not detected during BAC-end sequencing of the *Arabidopsis *genome suggesting it is not found as an isolated repeat. Rather, it is likely to be found embedded among the canonical variants at the tips of chromosomes 2 or 4, the two regions that harbor rDNA repeats. The frequency with which we have detected it in this study, 6.4% is surprisingly high. We have not yet ruled out the possibility of preferential amplification of the deleted variant because of its smaller size and simpler secondary structure. However, given estimates of 1200-1500 rDNA repeats, it is possible that multiple copies of this novel variant may be present in the *A. thaliana *diploid genome.

The existence of such a divergent 18 S sequence is at odds with highly homogenous sequences that might be expected from concerted evolution. Intra-individual polymorphic rDNAs may subsist either when concerted evolution is impaired by the location of rDNA repeats in non-homologous chromosomes, in polyploids and interspecific hybrids [14-19] or, when the rate of mutation exceeds that of concerted evolution [20,21]. Given the ancient polyploidy of the *Arabidopsis *genome [12] and the otherwise homogeneity of 18 S rDNAs observed, the presence of the deletion variant is unlikely to reflect impaired homogenization. Instead, it probably represents a relatively new mutation and repeats in transition stages of concerted evolution.

Our results show that *Arabidopsis thaliana *accessions are polymorphic for the presence of the deletion variant. *Arabidopsis *accessions generally exhibit a relatively high degree of polymorphism that is often shared worldwide; yet some population structure and isolation by distance is evident [22-24]. The availability of such large scale population data in *Arabidopsis *will enable studies on the inheritance pattern of the deletion variant and evolution of rDNA in *Arabidopsis*.

The extent of the observed deletion is unprecedented. Indels reported for rDNA coding regions within a species generally concern one or two nucleotides. The 270 bp deletion encompasses the universally conserved helix 18 or '530 loop', critical for ribosome function. Crystal structures of bacterial 30 S ribosomal subunit have established the role of helix 18 in decoding. Correct binding of mRNA and cognate tRNA in the A site of the ribosome induces conformational change of G530, which then interacts with the second position of the anticodon and the third position of the codon [25]. Analysis of 16 S mutants in bacteria corroborates the importance of helix 18. Typically, mutations in helix 18 result in a lethal phenotypes [26,27]. The importance of this region is also underscored by the fact that it is the target of several antibiotics.

Despite the fact that a functionally critical helix is missing, our results show that the deleted variant is expressed. Several studies in *Arabidopsis *have revealed that rDNA expression is modulated by a number of factors. Only a fraction of all the rDNA repeats are transcribed because of dosage compensation mechanisms that involve large scale silencing and a similar mechanism operates in silencing specific arrays in hybrids [28-30]. Additionally this large scale silencing is under the control of developmental switches as evidenced by onset during early stages of seed germination [31,32]. Studies focusing on 5 S rDNA have also shown that aberrant repeats are also silenced. Indeed, specific 5 S rRNA loci were shown to be methylated to prevent production of aberrant transcripts [32,33].

If misfolded, mutated or non-functional rRNA are transcribed, several quality control mechanisms lead to their degradation before or after ribosome biogenesis. These include the exosome mediated quality control of misfolded pre-rRNAs following their polyadenylation [34-37] as well as the 'non-functional rRNA decay' leading to decreased stability of the mature rRNA contained in fully assembled ribosomes and ribosomal subunits [38].

It is thus surprising that we have been able to detect the expression of a significantly truncated variant. The expression of the deleted variant appears to be under tight control in young seedlings and relaxed during later stages of development. Whether this control is strictly dependent on developmental stage or sequence is not clear. Thus, this finding opens up the possibility of investigating factors controlling the expression of such aberrant 18 S rRNAs in plants.

In conclusion, this study documents the existence of a new 18 S variant with a 270 bp deletion and demonstrates the incomplete homogenization of rDNA coding regions in *Arabidopsis thaliana*. Our results also show that *Arabidopsis *accessions are polymorphic for this variant, which open-up the possibility of investigating the evolution and inheritance pattern of aberrant rDNA variants in the context of studies that examine the population structure of *Arabidopsis*. In addition, its expression is dependent on the developmental stage of the plant, with tight control in seedlings suggesting that transcriptional or post-transcriptional silencing mechanisms are at play.

## Methods

For cloning 18 S rDNA, genomic DNA was extracted from *Arabidopsis thaliana *ecotype Columbia (Col-1) grown for 10 days under sterile conditions on MS media [39] using a modified CTAB method [40]. For cloning transcribed 18 S rDNA, total RNA was extracted from 100 mg plant material using a Plant RNAeasy kit (Qiagen) and treated with DNAseI (Invitrogen) according to the manufacturer's protocol. The total RNA was then reverse transcribed with random primers using the SuperScript RT-PCR system (Invitrogen).

18 S sequences were PCR amplified from two independent extracts using Platinum Pfx polymerase (Invitrogen) with FwdFull 5'CACC TACCTGGTTGATCCTGCCA3' and RevFull 5 'ATCCTTCCGCAGGTTCAC 3'primers. The primer pair amplifies a 1803 bp fragment from the 18 S rDNA, At3g41768 (GenBank ID: 186510611). The PCR enhancer reagent was included in the reaction. The PCR reaction was carried out for 30 cycles with an annealing temperature of 58°C for 30 seconds and an extension time of two and a half minutes at 68°C. The PCR product running at about 1.8 kb was excised from the gel, cleaned and cloned in the directional pENTR-D-TOPO vector (Invitrogen). The presence of an insert was verified by digesting plasmid preps with *Hpa*I and *Eco*RV.

A total of 47 clones generated from genomic DNA and 34 clones generated from cDNA were sequenced with universal FwdM13 and RevM13 as well as 18 S internal primers 5'TCGATGGTAGGATAGTGG3' and 5'ACATCTAAGGGCATCACA3' to cover the entire length of the insert. Trace files were imported in CodonCode Aligner V.3.0.1 (CodonCode Corp.); vector sequences removed and assembled using CAP3 [41]. Sequences were analyzed processed in CodonCode. Low quality bases (q < 20) were automatically replaced by that of the consensus sequence and all other discrepancies resolved manually. The level of polymorphism was calculated per fragment length (total number of clones deviating from the consensus/length of 18SrDNA which is 1808 bp) and per sequenced clones (total number of clones deviating from the consensus/total number of sequenced clones).

For the detection of the presence and expression of the deleted variant, genomic DNA and total RNA were extracted from *Arabidopsis thaliana *ecotype Columbia (Col-1) as above. RNA samples were obtained from 10 days old seedlings or roots of two weeks old plants grown under sterile conditions as well as mature leaves, stems and flowers from plants grown in soil for 5 weeks. For testing polymorphisms, DNA was extracted from ecotypes Landsberg erecta, Bay and Shahdara.

To specifically detect the presence of the deleted variant, the FwdFull primer as above was used with a new reverse primer, RevDel 5' AGGCACGACCCGGCCAGG 3'. The two primers amplify a 460 bp fragment exclusively from the deleted variant when a polymerase lacking proofreading activity is used. Hence, the Platinum SuperMix (Invitrogen) was used for the detection of the deleted variant. Amplification of eukaryotic translation initiation factor 4A fragments (At3G19760) served as internal controls. Primers FwdeIF4A: 5'TAGAAGAGGCGGTGGAGCTA 3' and ReveIF4A: 5'TCTGGTCCTTGAACCCTCTG 3'were designed such that amplification from genomic DNA would result in a 872 bp fragment and amplification from cDNA would result in a 560 bp fragment.

For visualization of the deleted region on the secondary structure of *Arabidopsis *18 S ribosomal RNA, the structure was downloaded from the RNA STRAND database [42] and edited using Inkscape version 0.48.0-1.

## Nucleotide sequence

The sequence of the 18 S variant with 270 bp deletion has been deposited in GenBank under the Accession No. GQ380689

## Competing interests

The authors declare that they have no competing interests.

## Authors' contributions

MJ amplified, cloned and prepared 18 S rDNA and rRNA for sequencing. RF carried out experiments to confirm the presence and expression of the 18 S deletion. AM conceived the experiments, analyzed sequences and wrote the manuscript.
